# Increased cytotoxicity and streptolysin O activity in group G streptococcal strains causing invasive tissue infections

**DOI:** 10.1038/srep16945

**Published:** 2015-11-25

**Authors:** Nikolai Siemens, Bård R. Kittang, Bhavya Chakrakodi, Oddvar Oppegaard, Linda Johansson, Trond Bruun, Haima Mylvaganam, INFECT Study Group, Per Arnell, Ole Hyldegaard, Michael Nekludov, Ylva Karlsson, Mattias Svensson, Steiner Skrede, Anna Norrby-Teglund

**Affiliations:** 1 Center for Infectious Medicine, Karolinska Institutet, Stockholm, Sweden; 2 Haraldsplass Deaconess Hospital, Bergen, Norway; 3 Department of Clinical Science, University of Bergen, Bergen, Norway; 4 Department of Medicine, Haukeland University Hospital, Bergen, Norway; 5 Department of Microbiology, Haukeland University Hospital, Bergen, Norway; 6 Department of Anaesthesiology and Intensive Care Medicine, Sahlgrenska University Hospital, Gothenburg, Sweden; 7 Department of Anaesthesia, Rigshospitalet, Copenhagen, Denmark; 8 Department of Physiology and Pharmacology, Section for Anesthesiology, Karolinska University Hospital, Stockholm, Sweden; 9 Department of Anesthesiology and Intensive Care, Blekinge Hospital, Karlskrona, Sweden

## Abstract

*Streptococcus dysgalactiae* subsp. *equisimilis* (SDSE) has emerged as an important cause of severe skin and soft tissue infections, but little is known of the pathogenic mechanisms underlying tissue pathology. Patient samples and a collection of invasive and non-invasive group G SDSE strains (n = 69) were analyzed with respect to virulence factor expression and cytotoxic or inflammatory effects on human cells and 3D skin tissue models. SDSE strains efficiently infected the 3D-skin model and severe tissue pathology, inflammatory responses and altered production of host structural framework proteins associated with epithelial barrier integrity were evident already at 8 hours post-infection. Invasive strains were significantly more cytotoxic towards keratinocytes and expressed higher Streptokinase and Streptolysin O (SLO) activities, as compared to non-invasive strains. The opposite was true for Streptolysin S (SLS). Fractionation and proteomic analysis of the cytotoxic fractions implicated SLO as a factor likely contributing to the keratinocyte cytotoxicity and tissue pathology. Analyses of patient tissue biopsies revealed massive bacterial load, high expression of *slo*, as well as immune cell infiltration and pro-inflammatory markers. Our findings suggest the contribution of SLO to epithelial cytotoxicity and tissue pathology in SDSE tissue infections.

In all ages, simple skin and soft tissue infections are common. In contrast, severe soft tissue infections are far less frequent, but yet represent a great health and resource burden. Necrotizing soft tissue infections (NSTIs) are the most extreme end of skin and soft tissue infections and the vast majority of NSTI patients require intensive care and extensive surgical interventions to remove necrotic tissue. Despite many improvements in medical care, the mortality associated with NSTIs has not changed during the last decades and remains above 20%^1^,^2^. Although a number of bacteria can cause NSTI, including gram-negative bacilli, *Staphylococcus aureus*, and *Clostridum spp*, the main causative microbe is group A streptococcus (GAS). However, *Streptococcus dysgalactiae subspecies equisimilis* (SDSE) has emerged as an important cause of invasive infections, in particular those associated with skin and soft tissue including NSTIs^3^,^4^,^5^,^6^,^7^.

SDSE is a commensal constituent of the human microbiota and was long considered less pathogenic than other streptococci^8^. However, several publications have reported invasive infections caused by beta-hemolytic SDSE, which almost exclusively belongs to group G or C streptococci (GGS, GCS)^5^,^9^,^10^,^11^. SDSE infections often present as superficial skin and soft tissue infections, including cellulitis and erysipelas. More rarely, life-threatening NSTIs and streptococcal toxic shock syndrome (STSS) may develop^12^,^13^,^14^. Primary focal infections are considered to be ports of entry leading to severe outcome^15^.

Certain GAS *emm*-types, particularly *emm1* and *emm3*, are over-represented in severe cases and have been linked to NSTI, STSS, and mortality^16^. In case of SDSE, there is no clear link between clinical severity and *emm*-types. Recent studies in the US and Norway suggested *stG643* as a dominant *emm*-type^17^,^18^, whereas this type was completely absent in a Japanese study^7^. According to several studies of invasive beta-hemolytic streptococcal infections, SDSE infected patients tended to be older and had more often underlying conditions than GAS patients^4^,^7^,^19^,^20^,^21^.

In NSTIs the rapid tissue destruction is proposed to involve toxin induced neutrophil aggregate-mediated vascular occlusion, followed by ischemia which expands until the tissue is destroyed^22^. In GAS NSTI several factors have been proposed to contribute to tissue pathology including among others superantigens, M1-protein, the cysteine protease SpeB, and a pathologic host inflammatory response^23^. However, data on the contribution of virulence factors to severe outcome of SDSE infections are scarce. SDSE are phylogenetically related to GAS and they share several virulence factors, including antiphagocytic M-Protein, Streptolysin S (SLS), Streptolysin O (SLO) and Streptokinase (Ska)^6^,^18^,^24^,^25^,^26^. Although SDSE strains may harbor superantigen genes, i.e. s*peG*^*dys*^, no superantigenic activity has been detected^27^.

We sought to deepen our insight into the pathogenesis of invasive SDSE infections with a particular focus on tissue pathology. To this end, a clinical group G SDSE strain collection from invasive and non-invasive cases was analyzed for virulence factor expression and their effect on human keratinocytes, immune cells, and in organotypic skin tissue. The results implicated SLO as a key factor mediating cytotoxicity and tissue pathology in SDSE skin and soft tissue infections.

## Results

### Bacterial dissemination and tissue pathology in organotypic skin models

**Figure 1:**
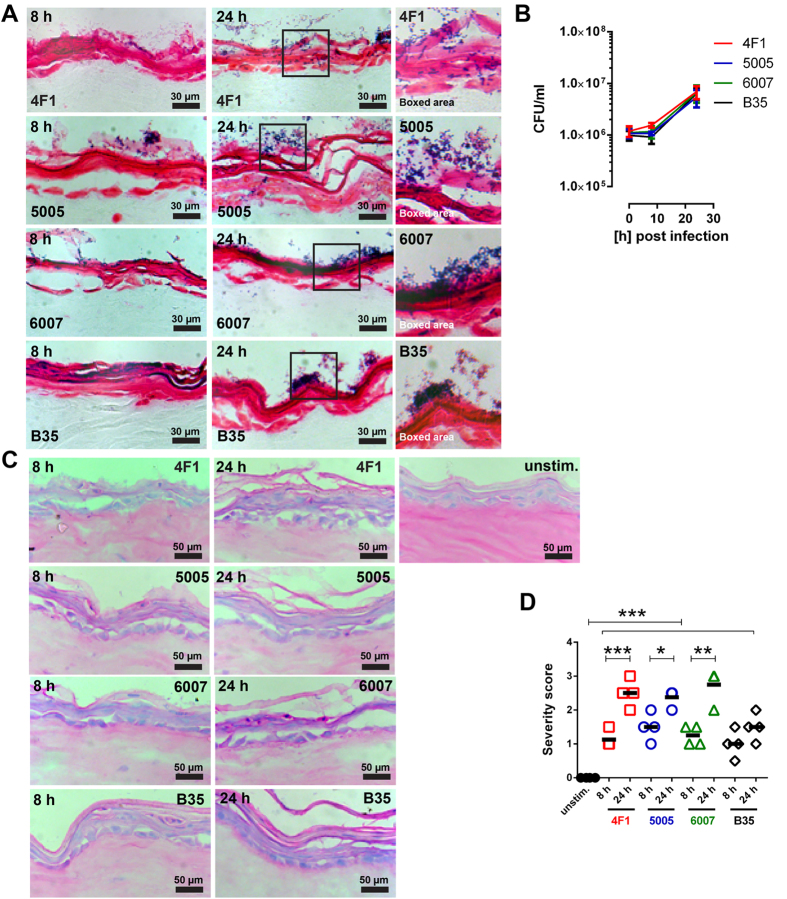
SDSE infections in skin tissue model. (**A**) Representative micrographs of Gram-stained infected tissue with indicated SDSE strains and total CFU counts (**B**) of bacteria recovered from skin tissue models. The data represent the mean values ± SD (n = 3). (**C**) Histological analysis of the skin model tissue after infection. Representative images with indicated time points post infection with indicated strains are shown. (**D**) Blinded scoring of tissue pathology of the skin model after infection. Horizontal lines denote median values. The statistical significance was determined using Kruskal-Wallis with Dunn´s multiple comparison test. (*p < 0.05; **p < 0.01; ***p < 0.001).

To compare the ability of strains to colonize and invade complex tissue, organotypic skin tissue with a stratified epithelium and a fibroblast dermal layer was created and infected with selected SDSE strains representing diverse types and different severity of infections. The strains were isolated from tissue or skin and included three invasive NSTI strains (4F1, 5005, and 6007) and one non-invasive strain (B35) derived from an uncomplicated wound infection. All four strains were able to colonize the tissue and bacterial replication was evident in all infected tissue (Fig. 1A,B). After eight hours of infection, the bacteria were found mainly associated with the apical side of the epidermis, while after 24 hours the invasive strains were frequently found in deeper epidermal layers whereas the non-invasive B35 strain remained associated with stratum corneum and did not disseminate to deeper layers (Fig. 1A).

Pathologic changes were assessed by microscopic analyses of hematoxylin/eosin stained tissue sections. This revealed that all three invasive strains caused severe tissue damage characterized by substantial epithelial disruption and detachment, which significantly increased over time (Fig. 1C,D). In contrast, B35 induced mild to moderate epithelial disruption (Fig. 1C,D).

**Figure 2:**
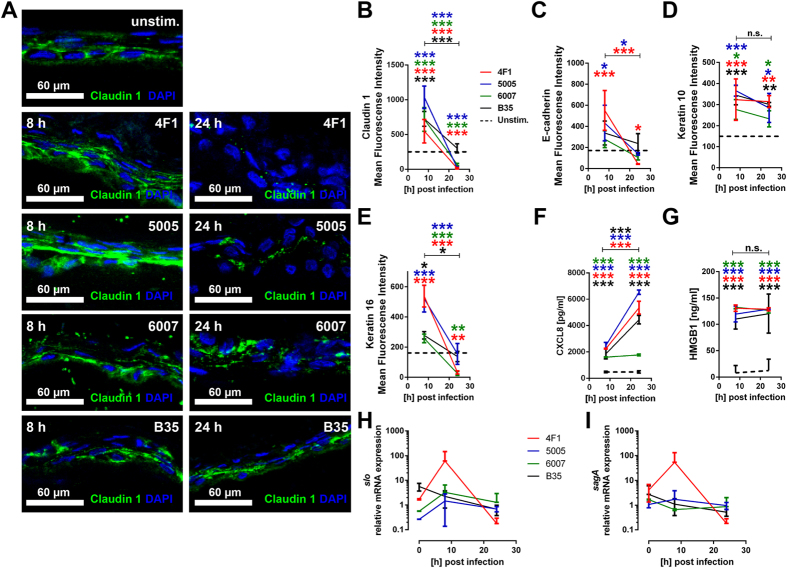
The impact of SDSE infection on epidermal tissue integrity and inflammation. (**A**) Representative immuno-staining of Claudin-1 and MFI (**B–E**) analysis of protein expression patterns of indicated proteins during the infection compared to the uninfected control (dashed line). Detection of CXCL8 (**F**) and HMGB1 (**G**) levels in model culture supernatants. (**H–I**) Relative mRNA expression of genes encoding for streptococcal virulence factors before (stationary phase cultures, t_0_) and during the infection in model tissue. Relative expression of *slo* and *sagA* before and during the infection are shown. The data represent the mean values ± SD (n ≥ 3). The statistical significance was determined using Kruskal-Wallis with Dunn´s multiple comparison test. (*p < 0.05; **p < 0.01; ***p < 0.001).

Next, the bacterial impact on epidermal structural proteins was examined by immuno-staining and confocal microscopy analysis. As illustrated in Fig. 2A–E, significantly increased expression of the junction proteins claudin-1 and E-cadherin as well as the keratins K10 and K16 was evident at 8 hours post-infection. As the infection progressed, the expression levels of most proteins, except K10, declined to that of the uninfected control or even below, likely due to the loss of tissue integrity (Fig. 2A,B,E).

Since NSTIs are characterized by hyper-inflammation and necrosis, we analyzed the supernatants of infected skin tissue models for CXCL8 and HMGB-1, the latter being a marker for both necrosis and inflammation^28^. Both CXCL8 and HMGB1 were significantly increased already after eight hours of infections with all four strains (Fig. 2F–G). While CXCL8 levels were increasing over time, the maximum of HMGB1 was reached already at 8 hours post-infection, indicating that the cell and tissue injury occurs at the early stage of infection.

By virtue of their cytotoxic activity, it seemed likely that SLO and/or SLS could be involved in the noted tissue injury. To this end, gene expression analysis of infected skin tissue was performed (Fig. H–I). Expression data of stationary phase cultures of the bacterial isolates was set as reference (t_0_). The results show that *slo* was strongly induced in the tissue milieu, and among the invasive strains, *slo* showed the strongest induction within 8 hours and then returned to the same level as the reference (Fig. 2H). In contrast, no changes in *sagA* expression, gene encoding for SLS, were found in the majority of infections (Fig. 2I). Only in the 4F1 strain, *sag*A expression showed a peak at 8 hours post-infection and then declined to the reference level.

### Cytotoxic effects towards keratinocytes

**Figure 3:**
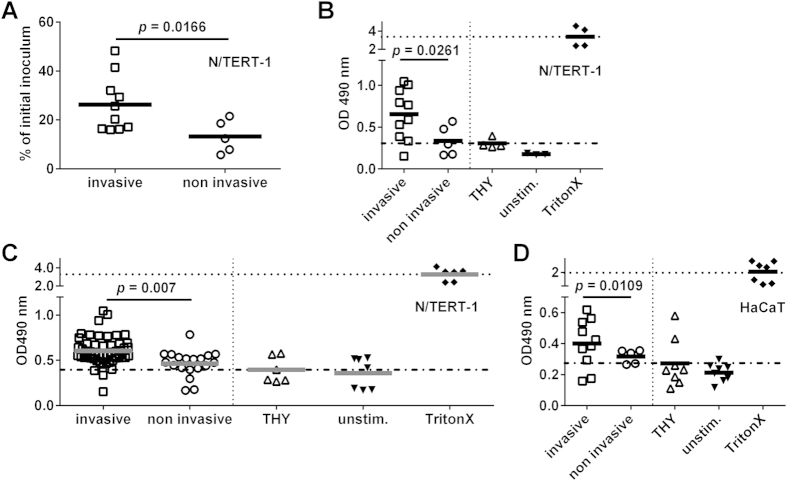
SDSE induced cytotoxicity towards human keratinocytes. (**A**) Total bacterial counts (selected cohort) recovered from N/TERT-1 cells 2 hours post infection and cytotoxicity towards these cells (**B**). (**C**) Cytotoxicity induced by bacterial supernatants from entire cohort on eukaryotic N/TERT-1 cells. (**D**) Cytotoxicity induced by bacterial supernatants from selected cohort on eukaryotic HaCaT cells. Each dot represents the mean vallue of three independent experiments with one strain. The horizontal lines within each group represent the mean value. The statistical significance between the two groups was determined using two-tailed Mann-Whitney U-test.

Taken together the results from infected skin models suggested that invasive SDSE strains were more cytotoxic resulting in greater tissue pathology. To further explore this finding, we analyzed the larger SDSE strain collection (Table S1) for cytotoxicity towards the epidermal keratinocyte cell line used to generate the skin models, i.e. N/TERT-1. First, live infections with selected strains were performed and infectivity as well as cytotoxicity was assessed (Fig. 3A,B). The results revealed that invasive SDSE strains had significantly higher infection rates and also resulted in higher cytotoxicity, as compared to non-invasive strains. Second, keratinocytes were stimulated with bacterial supernatants prepared from the entire strain collection (Table S1). Again supernatants from invasive strains elicited significantly higher cytotoxic effects as compared to non-invasive strains (Fig. 3C). Similar differences were seen using another keratinocyte cell line, HaCaT (Fig. 3D). In contrast, neither dermal fibroblasts or PBMC, nor primary neutrophils were affected by the bacterial supernatants (Fig. S1). Thus the data demonstrate that keratinocytes are particularly susceptible to cytotoxicity elicited by SDSE exotoxins.

### SLO mediated cytotoxicity towards keratinocytes

**Figure 4:**
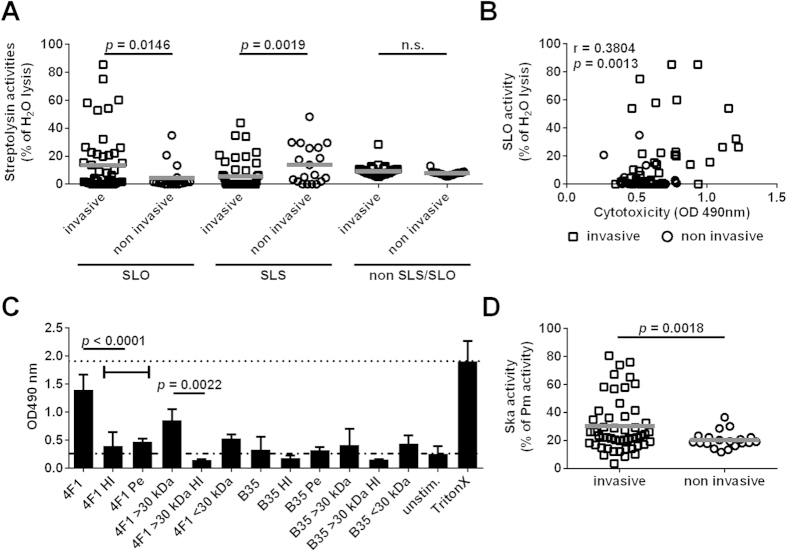
SLO-mediated cytotoxicity towards human keratinocytes. (**A**) Streptolysin activities in bacterial supernatants derived from stationary growth phase. Each dot represents the mean value of three independent experiments with one strain. The horizontal lines within each group represent the mean value. The statistical significance between the two groups was determined using two-tailed Mann-Whitney U-test. (**B**) Correlation analysis of SLO hemolytic activity and keratinocyte cytotoxic activity in bacterial supernatants. Correlation was determined using Pearson test. (**C**) Cytotoxicity induced by different fractions of bacterial supernatants from 4F1 and B35 strains (HI, heat inactivation; Pe, pepsin treatment). The data represent the mean values ± SD (n ≥ 3). The statistical significance between the two groups was determined using two-tailed Mann-Whitney U-test. (**D**) Streptokinase activities in bacterial supernatants derived from stationary growth phase. Each dot represents the mean value of three independent experiments with one strain. The horizontal lines within each group represent the mean value. The statistical significance between the two groups was determined using two-tailed Mann-Whitney U-test.

To explore whether streptolysins might be responsible for the noted cytotoxic effects towards keratinocytes, SLO, SLS as well as non SLO/SLS factors were assessed by a hemolytic assay in culture supernatants of all 69 clinical isolates. Significantly higher SLO activity was identified in invasive, as compared to non-invasive strains, whereas the reverse was true for SLS activity (Fig. 4A). Also, a hemolytic activity due to non SLO/SLS factors was measurable in all strains, but there was no difference between invasive and non-invasive (Fig. 4A). A positive correlation between SLO activity and keratinocyte cytotoxicity was found (Fig. 4B), whereas no such correlation was found with SLS or non SLS/SLO activity (Fig. S2).

Taken together, the data suggest that SLO might be responsible for the cytotoxicity towards keratinocytes. To further explore this, we performed fractionation, protein inactivation and proteomic analysis of cytotoxic fractions. For this purpose, we used two strains: one invasive (4F1) highly cytotoxic and one non-invasive (B35) eliciting lower cytotoxicity. Heat-inactivation or pepsin-treatment of the supernatants almost completely abolished the cytotoxic activity; thus, indicating the protein nature of the cytotoxic factor (Fig. 4C). Next, the supernatants were separated in two fractions with a 30 kDa cut off using filter ultra-centrifugation. The cytotoxic activity was predominantly found in the >30 kDa fraction from invasive 4F1 strain (Fig. 4C). B35 strain with less active SLO showed minor cytotoxic effects. Proteomic analysis of >30 kDa fractions from both 4F1 and B35 identified several virulence factors, including SLO, streptokinase, phosphoglycerate kinase (PGK), enolase (Eno), glyceraldehyde-3-phosphate dehydrogenase (GAPDH), and M protein. Notably, SLO was the only factor identified that had previously been ascribed cytolytic activity (Table 1 and Table S2). Since Streptokinase (Ska), a fibrinolytic factor secreted by streptococci, was also identified in >30 kDa fraction, we tested its activity in the entire cohort. This revealed significantly higher Ska activity in invasive strains compared to non-invasive (Fig. 4D).

### Streptolysins, immune cells and pro-inflammatory/necrotic markers in patient samples

**Figure 5:**
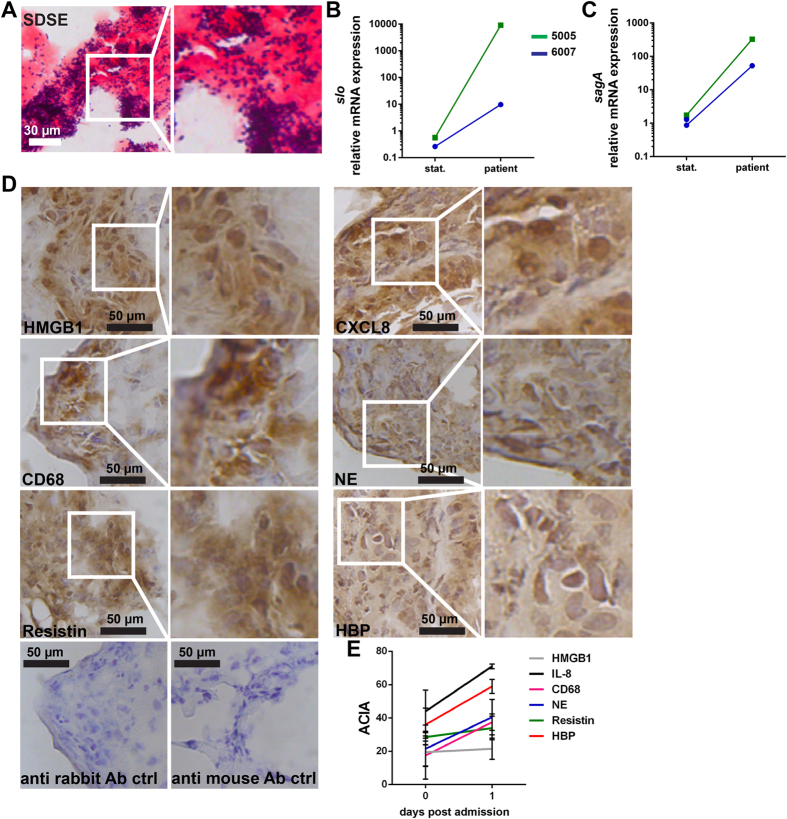
Infammation in patient biopsies as a result of high infiltration by bacteria and toxin production. (**A**) High bacterial load in patient biopsies visualized via Gram-staining. Relative mRNA expression of *slo* (**B**) and *sagA* (**C**) in patient biopsies. (**D**) Immunohistochemical detection of pro-inflammatory markers HMGB1 and CXCL,8 infiltrating immune cells (macrophages [CD68] and neutrophils [NE]), and activation markers Resistin and HBP in patient biopsies. (**E**) Image analysis values for all markers in patient biopsies at different days post admission. The sections were analyzed by acquired computerized image analysis (ACIA). Mean values ± SD from two biopsies of each patient are shown (dpa, days post admission).

Next, we analyzed tissue biopsies from the NSTI patients 5005 and 6007 to test the clinical relevance of our *in vitro* findings. Gram-staining detected massive bacterial load in the biopsies with Gram-positive chains, aggregations as well as single cocci found in all analyzed tissues (Fig. 5A). The gene expression of *slo* and *sag*A (SLS) in the day 0 tissue biopsies were determined, and in both patients the expression exceeded that of stationary phase *in vitro* bacterial cultures (Fig. 5B,C). In accordance with the results of infected skin tissue model, both HMGB1 and CXCL8 were readily detectable in the tissue biopsies (Fig. 5D,E). Also infiltrating phagocytic cells like macrophages (CD68) and neutrophils (NE), as well as the severity markers resistin and heparin binding protein (HBP) were present in the tissue (Fig. 5D). *In situ* image analysis of the stained tissue confirmed high levels of the markers in the tissue collected at both days 0 and 1 (Fig. 5E).

**Figure 6:**
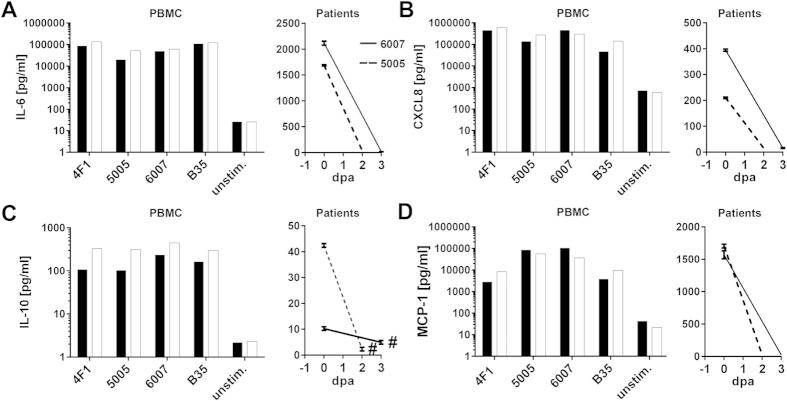
Cytokine release by human immune cells in response to SDSE supernatant stimulations. (**A**) IL-6, CXCL8 (**B**), IL-10 (**C**), and MCP-1 (**D**) release by PBMCs from two healthy donors after stimulation with supernatants from indicated strains (left panel) and in patient plasma samples (right panel). #, under detection limit. Mean values ± SD from two technical replica of each patient are shown (right panel).

Also plasma samples from patients 5005 and 6007 were analyzed for a panel of inflammatory markers, including CXCL8, IL-6, MCP-1, and IL-10 (Fig. 6A–D). The results revealed high levels of CXCL8, IL-6 and MCP-1 in day 0 samples. Also IL-10 was detected, although at lower levels. Similarly, stimulation of PBMCs with bacterial supernatants from strains 5005, 6007, 4F1, and B35 showed that all strains elicited high levels of CXCL8, IL-6, MCP-1, and somewhat lower IL-10 (Fig. 6A–D).

As superantigens are major mediators of systemic toxicity in GAS infections^29^,^30^ and the superantigen gene *speG*^*dys*^ was identified in the majority of the SDSE strains (Table S1), we tested the superantigenic activity of the SDSE strains. However, no proliferation of PBMC was triggered by any of the SDSE strains. Only the positive controls, PHA and GAS supernatant induced proliferation (Fig. S3).

## Discussion

Since the last decade an increasing burden of invasive infections, including also cases of NSTI and STSS, due to SDSE has been reported, and some countries even report a disease burden comparable to GAS^12^,^13^,^14^,^31^. Although SDSE share some of the virulence factors produced by GAS, such as superantigens (i.e. *speG*^*dys*^), M protein, SLO, SLS, and Ska^6^,^18^,^24^,^25^,^26^, little is known of their contribution to disease pathogenesis. Here we demonstrate that clinical group G SDSE strains efficiently infect human skin tissue and cause tissue pathology. Notably, severity of infection was related to keratinocyte cytotoxicity, epithelial damage in the skin tissue model, as well as SLO activity with increased activities noted in invasive as compared to non-invasive strains. Also, *slo* gene expression was found to be highly expressed in NSTI patient tissue biopsies. Additional analyses including fractionation and proteomics further implicated SLO as the cytotoxic factor responsible for the SDSE-mediated tissue pathology.

In *in vivo* murine experimental models, SLO has been reported as a factor contributing to GAS tissue infections^32^. In contrast, Humar and colleagues^5^ suggested that SLS contributed to NSTI via direct cytotoxicity to cells of the tissue and feeding vessels, leading to cell death and provoking neutrophil influx. This contradicts to our data in which no association with SLS activity and cytotoxicity to keratinocytes or skin tissue could be noted. One plausible explanation is that our study focuses on human cells, which may have different susceptibility to the streptolysins, as compared to murine cells used in^5^. Instead, our data supports a role for SLO in SDSE skin and soft tissue infections, in concordance with what was reported in GAS infections.

We are in this study focusing on tissue infection and for this purpose we have created and used organotypic skin tissue, which resembles real skin inasmuch as it provides a protective physiological barrier and consists of stratified epithelium and dermal layer containing fibroblasts. The SDSE strains, both invasive and non-invasive, could efficiently infect the skin tissue and showed similar massive bacterial load as evident in patient tissue biopsies. However, especially the invasive strains showed dissemination throughout the entire epithelium. As already discussed, these strains have higher SLO activity likely contributing to the greater tissue disruption, which might promote dissemination. In addition, the invasive strains had significantly higher Ska activity as compared to non-invasive strains. As Ska is known to promote invasion through plasmin activation and subsequent proteolysis, it is tempting to assume that also Ska could contribute to the noted increased bacterial dissemination. However, this warrants further investigations.

The SDSE infection, in both infected skin tissue and patient biopsies, resulted in high levels of the inflammatory markers CXCL8 and HMGB1, the latter of which is also associated with necrosis. This is in line with the recent report by Johansson *et al.*^28^, where HMGB1 was suggested to be a marker for severity of GAS NSTI. Also analyses in patient plasma identified high levels of pro-inflammatory factors and PBMCs responded strongly to SDSE bacterial supernatants with high cytokine production. The factors eliciting inflammatory responses are as of yet unknown, but there is no evidence, in this study nor in others, to support superantigenic activity.

Bruun and colleagues^4^ reported differences in clinical presentation of severe SDSE and GAS skin and soft tissues infections with the former being associated with anatomically more superficial presentation. This is in line with our data revealing SLO-mediated cytotoxicity of keratinocytes, rapid tissue injury in the epithelium of infected skin tissue, and a loss of tissue integrity with reduced claudin-1 and K16 expression.

To our knowledge, this is the first report that implicates SLO as a cytotoxic factor likely contributing to SDSE mediated severe skin and soft tissue infections. Further studies are warranted to test SLO-targeted interventions.

## Methods

### Patient samples and ethics statement

Snap-frozen tissue biopsies and plasma samples were collected from two group G SDSE NSTI patients (5005 and 6007) enrolled in the EU-funded project INFECT (www.fp7infect.eu) in Gothenburg (Sweden) and Bergen (Norway). Written informed consent was obtained from both patients. The studies were approved by the regional Ethical Review Boards in Gothenburg (Regionala etikprövningsnämnden, Göteborg) and in Bergen (Regionale komiteer for medisinsk og helsefaglig forskningsetikk Vest).

Blood samples from healthy volunteers or buffy coats of blood provided by the blood bank at the Karolinska University Hospital were used. The buffy coats were provided anonymously; hence informed consent was not necessary. In case of healthy volunteers, donors were individuals well acquainted with the research conducted; thus, verbal informed consent was deemed sufficient. The ethical research committee at Huddinge University Hospital (Forskningskommitté Syd) approved the study including the above specified consent procedure.

All experiments were carried out in full accordance with the approved ethics applications specified above.

### SDSE strains, human cells, and culture conditions

A collection of non-invasive (*n* = 19) and invasive (*n* = 50) group G SDSE isolates collected from patients identified at Haukeland University Hospital, Bergen, during the period from 2003 to 2013 was used^4^,^18^,^24^. All patients, except two, had tissue involvement of varying severity ranging from wound infection to NSTI. Clinical presentation, *emm* type and presence of *spe*G^dys^ are presented in Table S1. SDSE strains 5005 and 6007 were isolated from NSTI patients enrolled in the INFECT study during 2013. All SDSE strains were cultured in Todd Hewitt Broth (Invitrogen) supplemented with 1.5% (w/v) yeast extract (THY, Invitrogen) under 37 °C and 5% CO_2_ atmosphere. Cell-free supernatants were prepared from bacterial cultures by centrifugation at 4,000 × g, 10 minutes.

The human keratinocyte cell lines N/TERT-1 and HaCaT were cultured in EpiLife medium (Invitrogen) under 37 °C and 5% CO_2_ atmosphere.

Human neutrophils and PBMCs were isolated from peripheral blood collected from healthy donors by Polymorphprep or Ficoll-Hypaque gradient centrifugation (Axis-Shields), respectively. Both cell types were cultured in RPMI 1640 media (HyClone) supplemented with 5% (v/v) FCS.

### Strain typing

Extraction of chromosomal DNA, *emm*-typing, and detection of *speG*^*dys*^ were performed as previously described^4^,^18^.

### Streptolysin and streptokinase activity in bacterial supernatants

SLO, SLS and non SLO/SLS hemolytic activity was measured by use of the conventional erythrocyte lysis activity assay as previously published^33^ and the activities related to a water lysis control. THY was used as a negative control.

Ska activity was assessed by the chromogenic assay as previously published^34^. The activities were related to the activity of the plasmin control. THY was used as negative control.

### Adherence, bacterial supernatant stimulations, and cytotoxicity LDH release assays

Bacterial adherence to epithelial cells was quantified using the antibiotic protection assay^35^. Cells were infected with SDSE strains at a multiplicity of infection of 1:10. Total bacterial counts after 2 hours of infection were determined by plating serial dilutions on blood agar plates. The results are shown as % of initial inoculum.

Cells were stimulated with bacterial supernatants (1:50 diluted). Keratinocytes (1.5 × 10^5^) and PBMCs (8 × 10^5^) were stimulated for 24 hours and neutrophils (8 × 10^5^) for 2 h after which supernatants were collected. THY and TritonX-lysed cells were used as negative and positive controls, resp.

Cytotoxicity was determined by measurement of the LDH activity via CytoTox 96 Non-Radio Kit (Promega) according to manufacturer’s guidelines.

### Cell proliferation assay

PBMCs (2.5 × 10^5^ cells/well) were seeded in 96-well plates and stimulated for 72 h with varying dilutions of bacterial supernatants (1:10–1:1000). The cells were pulsed with 1 μCi of ^3^H-thymidine (GE Healthcare) for additional 6 h of incubation and the uptake of the ^3^H-thymidine was measured.

### 3D organotypic skin model

The organotypic tissue was generated following the protocol of established lung tissue model with minor modifications^36^. Briefly, dermal equivalents were generated by adding 1 ml cell-free collagen (1 mg/ml; Advanced Biomatrix) into a six well-filter insert (Corning Life Sciences). After polymerization, 3 ml of NHDF populated (0.4 × 10^5^) collagen (2 mg/ml) was pipetted onto the polymerized collagen layer. After polymerization, dermal equivalents were submerged in DMEM medium for one week. After 7 days, 1 × 10^6^ N/TERT-1 cells were seeded onto the dermal equivalent and incubated for 2 h at 37 °C under a 5% CO_2_ atmosphere after which 2 ml of EpiLife (Life Technologies) was added to each insert. Submerged cultures were incubated for additional 3 days. Then skin models were air exposed by removing the medium from the inserts and adding 10 ml 1.46 mM CaCl_2_-EpiLife to the outer chamber. Air-exposed skin models were incubated for up-to 7 d at 37 °C under a 5% CO_2_ atmosphere, and the culture media was replaced every second day in the outer chamber. The models were infected with an optimal infectious dose of 1 × 10^6^ bacteria for 8 and 24 h. Total bacterial counts (extra- and intracellular bacteria) from model tissue, which was digested with Collagenase I (Roche) and sonicated, were determined by plating serial dilution on blood agar plates.

### RNA Preparation and Quantitative Reverse Transcription PCR Analysis (qRT-PCR)

Bacterial RNA was isolated using FastRNA Blue (MP Biomedicals) according to manufacturer´s guidelines. cDNA synthesis was performed using the Superscript first-strand synthesis system for RT-PCR (Invitrogen). The real-time PCR amplification was performed with SYBR GreenER Kit (Invitrogen) using an ABI Prism 7500 sequence detection system (Applied Biosystems). The level of *gyrA* transcription was used for normalization of bacterial genes. The primers used in this study are published by Watanabe *et al.*^33^.

### Protein extraction and digestion

Concentrated >30 kDa fractions of bacterial supernatants were re-suspended in 0.1% ProteaseMax (Promega), 50 mM ammonium bicarbonate and 10% acetonitrile. Protein concentrations were determined using the BCA kit (Pierce). 5 μg of each sample were incubated for 30 min at 50 °C followed by 10 min bath sonication at room temperature. Samples were centrifuged and the supernatant was directly subjected to a tryptic digestion protocol carried out by a liquid handling robot (MultiProbe II, Perkin Elmer).

### Liquid chromatography tandem mass spectrometry

Tryptic peptides were cleaned with C18 StageTips (Thermo Scientific) and the resulting peptide mixture was injected into an Ultimate system (Thermo Scientific) in-line coupled to a Q Exactive mass spectrometer (Thermo Scientific). The chromatographic separation of the peptides was achieved using an in-house packed column (C18-AQ ReproSil-Pur®, Dr. Maisch GmbH). The MS acquisition method was comprised of one survey full scan ranging from m/z 300 to m/z 1650 acquired with a resolution of R = 70’000 at m/z 400, followed by data-dependent HCD scans from maximum ten most intense precursor ions.

### Protein identification and quantitation

Tandem mass spectra were extracted using Raw2MGF (in-house software), and the resulting mascot generic files were searched against the firmicutes concatenated SwissProt protein database using the Mascot Deamon 2.4.1 search engine (Matrix Science Ltd.). Parameters were chosen as follows: up to two missed cleavage sites for trypsin, peptide mass tolerance 10 ppm, 0.05 Da for the HCD fragment ions. Carbamidomethylation of cysteine was specified as a fixed modification, whereas oxidation of methionine and deamidation of asparagine and glutamine were defined as variable modifications.

### Histological analysis and immunostaining of tissue biopsies

For cryosectioning, skin tissue models were treated with 2.0 M sucrose for 1 hour before embedding in optimum cutting temperature compound (Sakura Finetek) followed by freezing in liquid nitrogen and stored at −80 °C. 8 μm cryosections were obtained using a MICROM cryostat HM 560 MV (Carl Zeiss) and fixed in 2% freshly prepared formaldehyde in PBS for 15 minutes at room temperature or in ice-cold acetone for 2 minutes at −20 °C.

For histological analysis, the sections were stained for 15 seconds in Mayer’s haematoxylin and counterstained for 2 minutes in eosin. Histological severity scoring was performed in a double blinded manner using the following criteria, 0 unaffected tissue, 0.5–1 mild injury with minor epithelial loosening, 1.5–2 moderate injury with some epithelial disruption, 2.5–3 severe injury with continuous epithelial disruption and some detachment, 4 = extensive injury, massive epithelial disruption and detachment. SDSE were identified via conventional Gram staining.

For immune analysis, skin models and patient biopsies were cryosectioned (8 μm), fixed in 2% (v/v) formaldehyde and immunostained as previously described^28^. The following antibodies were used for immuno-histochemistry: anti-human HMGB1 (Abcam), anti-human IL8/NAP-1 (Invitrogen), anti-human resistin (R&D systems), anti-human CD68/EBM11 (DAKO), anti-human NE/NP57 (DAKO), and anti-human HBP (DAKO). Biotinylated secondary antibodies included goat-anti-mouse IgG and goat-anti-rabbit IgG (both from Vector Laboratories). The immunohistochemically stained sections were analyzed by acquired computerized image analysis (ACIA)^28^. In brief, the cell area was defined by the hematoxylin counterstaining and the results are presented as percent positively stained area × mean intensity of positive staining. The following antibodies were used for immuno-fluorescence: anti-human claudin 1, anti-human cytokeratin 10, and anti-human cytokeratin 16 (all Abcam), anti-human E-cadherin (Invitrogen), anti-human fibronectin (Sigma). Specific staining was detected by Alexa 488-conjugated goat anti-mouse IgG and Alexa 488-conjugated goat anti-rabbit IgG (both from Molecular Probes). The stainings were visualized using a Nikon A1 confocal microscope (Nikon Instruments). The mean fluorescence intensity (MFI) in tissue sections was determined using NIS element AR image analysis software (Nikon).

### Bio-Plex Pro multiplex immunoassay

Levels of IL-6, CXCL8, IL-10, TNFα, and MCP-1 in patient plasma or PBMC supernatants were determined using a Bio-Plex Pro Human Cytokine Assay Kit (Bio-Rad) and the MagPix instrument (Luminex) according to manufacturers’ guidelines.

### CXCL8 and HMGB1 Enzyme-linked Immunosorbent Assay (ELISA)

The culture media from unstimulated and infected models were collected and stored at −20 °C until used. HMGB1 was quantified using a HMGB1 ELISA Kit (IBL International), and CXCL8 was determined using Il-8 Quantikine ELISA (R&D Systems) according to manufacturer´s guidelines.

### Statistical analysis

The significance of differences in cytotoxicity, adherence, and toxin activity assays was determined using the non-parametric Mann-Whitney U-test, two-tailed. The significance of differences between skin model samples in protein expression and CXCL8 and HMGB1 release was determined using non-parametric ANOVA, i.e. Kruskal-Wallis with Dunn´s multiple comparison test. Correlation analyses of SLO, SLS, and non-SLO/SLS hemolytic activities and keratinocyte cytotoxicity were determined using Pearson test. All statistical analyses were performed using Prism version 6 software (GraphPad). *P* < 0.05 was defined as statistically significant.

## Additional Information

**How to cite this article**: Siemens, N. *et al.* Increased cytotoxicity and streptolysin O activity in group G streptococcal strains causing invasive tissue infections. *Sci. Rep.* **5**, 16945; doi: 10.1038/srep16945 (2015).

## Supplementary Material

Supplementary Information

## Figures and Tables

**Table 1:** Virulence factors detected* in >30 kDa fractions of 4F1 and B35 strains.

Category	4F1 >30 kDa	B35 >30 kDa
Secreted	Streptolysin O	Streptolysin O
	Streptokinase	Streptokinase
Membrane associated	M-Protein	M-Protein
	Enolase	Enolase
	GAPDH	GAPDH
	PGK	PGK
	M-Protein	M-Protein
	PAM	

^*^Based on mass spectrometry analyses (GAPDH, glyceraldehyde-3-phosphate dehydrogenase; PGK, phosphoglycerate kinase; PAM, plasminogen binding group A streptococcal M-protein).
