# Supplementary material for: Increased cytotoxicity and streptolysin O activity in group G streptococcal strains causing invasive tissue infections
Source: Sci Rep. 2015 Nov 25;5:16945. doi: 10.1038/srep16945 (PMC4658506; doi:10.1038/srep16945)
Supplement: Supplementary Information [file srep16945-s1.pdf]

Supplementary Material

Increased cytotoxicity and streptolysin O activity in group G streptococcal strains causing invasive tissue infections

Nikolai Siemens[1], Bård R. Kittang[2,3], Bhavya Chakrakodi[1], Oddvar Oppegaard[4], Linda Johansson[1], Trond Bruun[3,4], INFECT Study Group, Mattias Svensson[1], Steiner Skrede[3,4], and Anna Norrby-Teglund[1*]

Table S1. Strains used in this study[a]

Disease category	Strain	Site of isolation	Clinical syndrome	Bacteremia	Sepsis	STSS	*emm type*	*speG[dys]*
invasive	4F1[b]	Soft tissue	NSTI	0	1	0	*stG6.3*	1
	3NF5[b]	Blood	NSTI	1	1	1	*stG10.0*	1
	3G7[b]	Blood	NSTI	1	1	1	*stG2574.0*	1
	S19[b]	Soft tissue	NSTI	0	1	0	*stC74a.0*	1
	S76[b]	Soft tissue	NSTI	1	0	1	*stG2078.0*	0
	5005[b]	Soft tissue	NSTI	0	1	0	*stG647.1*	1
	6007[b]	Soft tissue	NSTI	0	1	0	*stG2078.2*	0
	S7	Blood	Erysipelas/cellulitis	1	0	0	*stG643.1*	1
	S8	Blood	Erysipelas/cellulitis	1	0	1	*stG480.0*	1
	S9[b]	Blood	Erysipelas/cellulitis	1	1	0	*stG6.5*	0

S41	Blood	Erysipelas/cellulitis	1	0	0	*stG6.1*	0
S47	Blood	Erysipelas/cellulitis	1	0	0	*stG485.0*	1
S52	Blood	Erysipelas/cellulitis	1	0	0	*stG6.0*	1
S53	Blood	Erysipelas/cellulitis	1	0	0	*stG652.0*	1
S54	Blood	Erysipelas/cellulitis	1	1	0	*stC5345.1*	0
S59	Blood	Erysipelas/cellulitis	1	0	0	*stG245.0*	0
S60	Blood	Erysipelas/cellulitis	1	1	0	*stG480.0*	1
S62	Blood	Erysipelas/cellulitis	1	1	0	*stG643.1*	1
S70	Blood	Erysipelas/cellulitis	1	1	0	*stG643.0*	0
S73	Blood	Erysipelas/cellulitis	1	0	0	*stG6.0*	1
S88	Blood	Erysipelas/cellulitis	1	0	0	*stC74a.0*	1
S90	Blood	Erysipelas/cellulitis	1	1	0	*stG485.0*	1
S94	Blood	Erysipelas/cellulitis	1	0	0	*stG643.0*	0
S98	Blood	Erysipelas/cellulitis	1	1	0	*stG485.0*	1
S103	Blood	Erysipelas/cellulitis	1	0	0	*stG6.1*	0
S106	Blood	Erysipelas/cellulitis	1	0	0	*stG652.0*	1
S 114	Blood	Erysipelas/cellulitis	1	0	0	*stG652.0*	1
S 115[b]	Blood	Erysipelas/cellulitis	1	0	0	*stG2078.0*	0
S 117[b]	Blood	Erysipelas/cellulitis	1	1	0	*stG2078.0*	0
S 118	Soft tissue	Erysipelas/cellulitis	0	1	0	*stG10.0*	1
S58	Blood	Spondylodiscitis	1	1	0	*stG62647.0*	1

	S68	Blood	Wound infection, endocarditiis	1	0	0	*stG6.0*	1
	S86	Synovial fluid	Acute monoarthritis	0	0	0	*stG643.1*	1
	S96	Bone	Spondylodiscitis, epidural abscess	0	0	0	*stG485.2*	1
	S97	Blood	Septic bursitits, endophtalmitis	1	1	0	*stC74a.0*	1
	S102	Blood	Endocarditis	1	1	0	*stC74a.0*	1
	S20	Blood	Tenovaginitis	1	0	0	*stG6792.0*	1
	S25	Blood	Wound infection	1	0	1	*stG485.0*	1
	S11	Blood	Acute monoarthritis	1	0	0	*stG485.0*	1
	S57	Blood	Primary bacteremia	1	1	0	*stG5420.0*	0
	S 121	Blood	Wound infection	1	1	0	*stG643.0*	0
	S 126	Blood	Wound infection	1	1	0	*stG643.0*	0
	S 128	Blood	Primary bacteremia	1	1	0	*stG10.0*	1
	S 130	Blood	Bacteremia	1	1	0	*stG643.0*	0
	S 131	Blood	Erysipelas/cellulitis	1	0	0	*stG643.0*	1
	S 132	Blood	Primary bacteremia	1	1	0	*stG6.0*	1
	S 135	Blood	Inguinal abscess	1	0	0	*stG10.0*	1
	S 137	Blood	Erysipelas/cellulitis	1	1	0	*stG643.1*	1
	S 138	Blood	Primary bacteremia	1	1	0	*stG485.0*	1
non invasive	B8[b]	Skin	Wound infection	0	0	0	*stG485.0*	1
	B9[b]	Skin	Wound infection	0	0	0	*stG485.0*	1
	B10[b]	Skin	Wound infection	0	0	0	*stG6.0*	1

B31[b]	Skin	Wound infection	0	0	0	*stG652.0*	1
B35[b]	Skin	Wound infection	0	0	0	*stG480.0*	1
B69	Skin	Wound infection	0	0	0	*stG10.0*	1
B71	Skin	Wound infection	0	0	0	*stG643.0*	0
B81	Skin	Wound infection	0	0	0	*stG6.7*	0
B88	Skin	Wound infection	0	0	0	*stG643.1*	1
B89	Skin	Wound infection	0	0	0	*stG6.0*	1
B91	Skin	Wound infection	0	0	0	*stG62647.0*	1
B131	Skin	Wound infection	0	0	0	*stG6.0*	1
B135	Throat	Tonsillitis	0	0	0	*stG6.1*	0
B141	Skin	Wound infection	0	0	0	*stG6.4*	0
B143	Skin	Impetigo	0	0	0	*stG652.0*	1
B153	Skin	Wound infection	0	0	0	*stG6.3*	0
B163	Skin	Wound infection	0	0	0	*stG643.1*	1
B196	Skin	Impetigo	0	0	0	*stG485.0*	1

[a], SDSE strain collection was previously published by Kittang et al. and Bruun et al [1,2] except strains 5005 and 6007. [b], selected cohort

Table S2. Proteins identified in >30 kDa fraction from 4F1 and B35 strains

Accession	Title	Score	MW	Cover
**4F1**				
ENO_STRE4	Enolase OS=Streptococcus equi subsp. equi (strain 4047) GN=eno PE=3 SV=1	317	47269	31.5
SPG2_STRSG	Immunoglobulin G-binding protein G OS=Streptococcus sp. group G GN=spg PE=1 SV=1	232	63253	13.3
STRP_STREQ	Streptokinase C OS=Streptococcus dysgalactiae subsp. equisimilis GN=skc PE=1 SV=1	196	50109	35.7
G3P_STREQ	Glyceraldehyde-3-phosphate dehydrogenase OS=Streptococcus dysgalactiae subsp. equisimilis GN=gap PE=3 SV=2	147	35962	41.4
STRP_STRP1	Streptokinase A OS=Streptococcus pyogenes serotype M1 GN=ska PE=4 SV=2	132	49894	20
ALF_STRP3	Fructose-bisphosphate aldolase OS=Streptococcus pyogenes serotype M3 (strain ATCC BAA-595 / MGAS315) GN=fba PE=3 SV=1	130	31302	34.1
TACY_STREQ	Streptolysin O OS=Streptococcus dysgalactiae subsp. equisimilis GN=slo PE=3 SV=1	126	64010	36.2
M12_STRPY	M protein, serotype 12 (Fragment) OS=Streptococcus pyogenes GN=emm12 PE=1 SV=1	83	62866	5.9
PAM_STRPY	Plasminogen-binding group A streptococcal M-like protein PAM (Fragment) OS=Streptococcus pyogenes GN=pam PE=1 SV=1	83	43603	8.5
EFTS_STRP1	Elongation factor Ts OS=Streptococcus pyogenes serotype M1 GN=tsf PE=3 SV=1	61	37204	11
METE_STAHJ	5-methyltetrahydropteroyltriglutamate--homocysteine methyltransferase OS=Staphylococcus haemolyticus (strain JCSC1435) GN=metE PE=3 SV=1	51	85511	3.6
EFTU_STRP8	Elongation factor Tu OS=Streptococcus pyogenes serotype M18 (strain MGAS8232) GN=tuf PE=3 SV=1	45	43886	25.9
PGK_STRA1	Phosphoglycerate kinase OS=Streptococcus agalactiae serotype Ia (strain ATCC 27591 / A909 / CDC SS700) GN=pgk PE=3 SV=1	45	42088	16.3
TIG_STRU0	Trigger factor OS=Streptococcus uberis (strain ATCC BAA-854 / 0140J) GN=tig PE=3 SV=1	41	47289	4.9

ATPA_STRT1	ATP synthase subunit alpha OS=Streptococcus thermophilus (strain CNRZ 1066) GN=atpA PE=3 SV=1	33	54617	3.4
ACON_STRMU	Aconitate hydratase OS=Streptococcus mutans serotype c (strain ATCC 700610 / UA159) GN=acn PE=3 SV=2	33	98278	0.8
**B35**				
STRP_STRS1	Streptokinase G OS=Streptococcus sp. (strain 19909) GN=skg PE=4 SV=1	410	50168	40.9
SPG1_STRSG	Immunoglobulin G-binding protein G OS=Streptococcus sp. group G GN=spg PE=1 SV=1	282	47538	19.6
TACY_STREQ	Streptolysin O OS=Streptococcus dysgalactiae subsp. equisimilis GN=slo PE=3 SV=1	254	64010	36.1
ENO_STRE4	Enolase OS=Streptococcus equi subsp. equi (strain 4047) GN=eno PE=3 SV=1	157	47269	23
ALF_STRP1	Fructose-bisphosphate aldolase OS=Streptococcus pyogenes serotype M1 GN=fba PE=3 SV=2	156	31303	27.6
G3P_STRP1	Glyceraldehyde-3-phosphate dehydrogenase OS=Streptococcus pyogenes serotype M1 GN=gap PE=3 SV=2	135	36034	50.9
PGK_STRP3	Phosphoglycerate kinase OS=Streptococcus pyogenes serotype M3 (strain ATCC BAA-595 / MGAS315) GN=pgk PE=3 SV=1	78	42104	12.1
EFTU_STRP8	Elongation factor Tu OS=Streptococcus pyogenes serotype M18 (strain MGAS8232) GN=tuf PE=3 SV=1	50	43886	31.7
M12_STRPY	M protein, serotype 12 (Fragment) OS=Streptococcus pyogenes GN=emm12 PE=1 SV=1	47	62866	2.8

MW, molecular weight

Figure S1

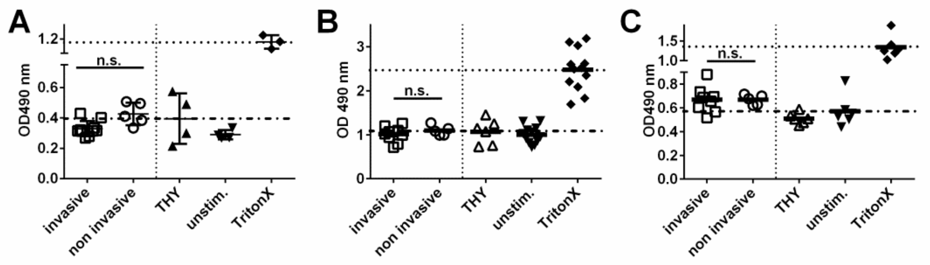

Figure S1. Cytotoxicity towards human primary fibroblasts (A), PBMCs (B), and PMNs (C). Human cells were stimulated with supernatants from selected cohort. The statistical significance between the two groups was determined using two-tailed Mann-Whitney U-test.

Figure S2

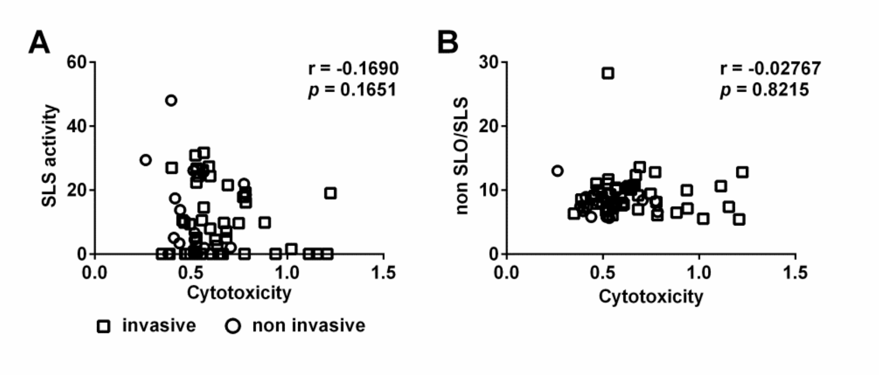

Figure S2. Cytotoxicity towards N/TERT-1 cells. (A) Correlation analyses of SLS and non SLO/SLS (B) mediated cytotoxicity towards human keratinocytes. Correlation was determined using Pearson test.

Figure S3

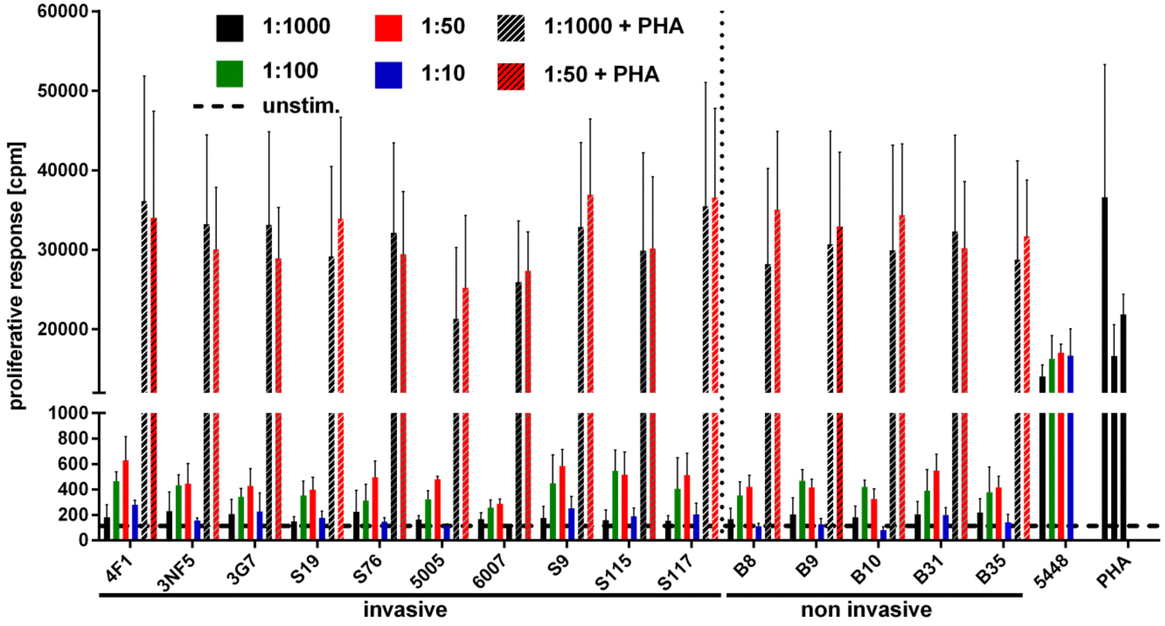

Figure S3. T-cell proliferation in human PBMCs. Proliferation was assessed by ^{3}H-thymidine uptake. Each bar represents the mean value ±SD from three donors.

REFERENCES

1 Bruun, T. *et al.* Necrotizing soft tissue infections caused by Streptococcus pyogenes and Streptococcus dysgalactiae subsp. equisimilis of groups C and G in western Norway. *Clin Microbiol Infect* **19**, E545-550, doi:10.1111/1469-0691.12276 (2013).

2 Kittang, B. R. *et al.* Invasive group A, C and G streptococcal disease in western Norway: virulence gene profiles, clinical features and outcomes. *Clin Microbiol Infect* **17**, 358-364, doi:10.1111/j.1469-0691.2010.03253.x (2011).
